# Openness and visibility of fungal bio(techno)logy

**DOI:** 10.1186/s40694-017-0038-x

**Published:** 2017-10-23

**Authors:** Vera Meyer, Corrado Nai, Alexander Idnurm

**Affiliations:** 1 Department of Applied and Molecular Microbiology, Institute of Biotechnology, Technische Universität Berlin, Gustav-Meyer-Allee 25, 13355 Berlin, Germany; 2 Federation of the European Microbiological Societies (FEMS), Delftechpark 37a, 2628 XJ Delft, The Netherlands; 3 School of BioSciences, The University of Melbourne, Building 122, Parkville, VIC 3010 Australia

Like a toddler having gone through its first phase of growth and development and now focusing more on developing its communication skills, *Fungal Biology and Biotechnology* is turning three this month—and exciting news lies ahead. Most notably, we are very happy to announce to past and future authors that since the end of September 2017 the journal *Fungal Biology and Biotechnology* is listed on PubMed/NCBI, which will have a positive effect on the reach, visibility, and impact of the studies published in the journal.

**Fig. 1:**
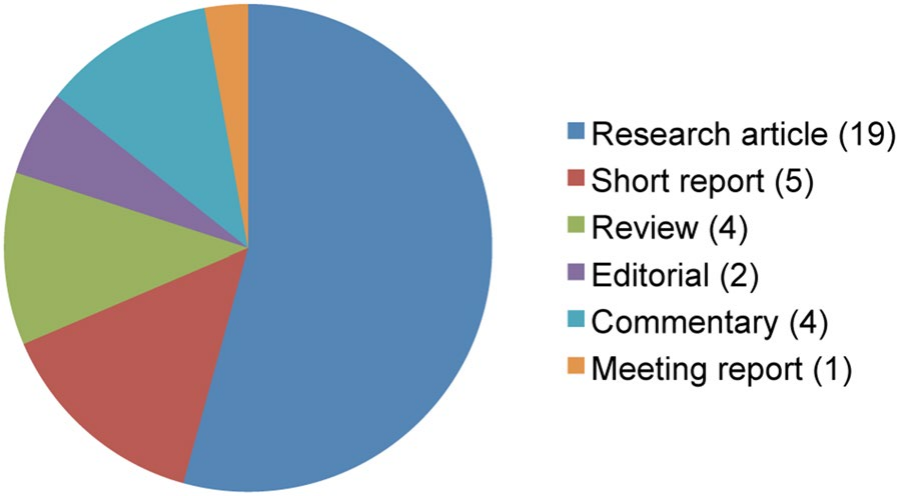
Portfolio of articles published by *Fungal Biology and Biotechnology* in its first 3 years of existence, as of October 9th 2017

After the first influential articles [1–4] going online on October 14th, 2014, the journal has published 28 original research and review articles that went through a rigorous peer-reviewing process (about 50% of submitted articles pass peer review). Beside original research articles and reviews, 4 commentaries, 2 editorials, and 1 meeting report were published as well (Fig. 1). Metric-based analyses consolidate and accentuate the trend reported in the editorial of October 2016 [5], with over 100,000 total accesses (average per article ca. 3000) and over 150 citations for the 35 articles. In a nutshell, *Fungal Biology and Biotechnology* is doing well and is prospering.

The articles in *Fungal Biology and Biotechnology* are shared and discussed within and outside the community of fungal bio(techno)logists, as testified by solid Altmetric [6] data (average Altmetric score per article over 5, with a peak of 61 for a recent commentary presenting the neglected intersection of fungi with the arts [7]), with the bulk of shares through Twitter. This interest is mirrored by the constant and steady increase of followers on the Twitter account of the journal, which are now more than 750. Corrado Nai regularly tweets via our *@FBBiotech* account about the hottest and newest findings in the field of fungal biology—and this of course includes also articles published by other research journals—to keep researchers updated and foster networking among them. We hope that by this activity *Fungal Biology and Biotechnology* can serve the fungal community to share knowledge among the different research sub-communities. As such, we encourage fellow researchers to take a look at *@FBBiotech* and to use the platform to follow and connect with the community.

In addition to social media being used to highlight the research within the journal and through *@FBBiotech* mycological research more widely, *Fungal Biology and Biotechnology* has actively used the BioMed Central blog to promote the cutting-edge research that is being conducted by the rising stars of the field. Three researchers, their research questions and their discoveries were thus featured in this blog after the 13th European Conference on Fungal Genetics in Paris (2016) and the 29th Fungal Genetics Conference at the Asilomar Conference Grounds in the United States (2017).

*Fungal Biology and Biotechnology* remains the first and only scholarly journal specifically devoted to cover the topic of fungal biotechnology, with all the content fully accessible for free to everyone (gold open access), as showed by a survey of some of the most influential or most recent journals focusing on fungi and/or biotechnology (excluding those publishing only reviews; Table 1). Although the scope of the different journals and the topics covered are often broad and overlapping among each other, *Fungal Biology and Biotechnology* succeeds in maintaining a specific niche while following the vision outlined in our kick-off editorial of 2014: “[*Fungal Biology and Biotechnology*] shall become a platform for scientists from academia and industry to present their hottest findings in unicellular or multicellular fungal systems, in medical or industrial strains, and in so far unexplored species. This will be a platform for experts to discuss their visions on how fungi can help us to address some of the key challenges of the twenty-first century.” [1] And not that the range of topics of the articles published so far is limited either—original research has focused on fungi as diverse as the model (industrial) species *Neurospora crassa*, *Aspergillus niger* and *Aspergillus nidulans*; plant pathogens as *Alternaria brassicicola*, *Botrytis cinerea, Claviceps purpurea*, *Leptosphaeria maculans*, *Puccinia* spp. and *Ustilago* spp.; basidiomycetes as *Sporobolomyces* sp. (yeast) as well as *Pleurotus sapidus* and *Hericium erinaceus* (edible mushrooms); further biotechnology- and ecologically-relevant species as *Penicillium nalgiovense* and *Trichoderma* spp.; and the slime mold *Physarum polycephalum*. The approaches used span molecular techniques (CRISPR/Cas9-driven genome editing, next-generation DNA and RNA sequencing, genotyping of sexual spores), screening of strains and strain-specific bioengineering, characterization of fungal populations, mathematical modelling of filamentous growth and intra-hyphal organelle movement, and a geographic information system to estimate filamentous growth on solid substratum. Overall, the studies [8] touch both basic and applied research and have relevance for areas as diverse as the industrial production of (bioactive) metabolites, the description of new antibacterial and antifungal substances, the analysis of the effect of fungal volatile organic compounds (VOCs) on plant growth, or the understanding of cell-wall stress/biogenesis and metal homeostasis in fungi.

**Table 1:** Examples of journals covering topics of fungal biology and/or biotechnology

Journal title	Publisher	Publishing model	Main research focus
*FEMS Yeast Research*	Oxford University Press	Hybrid	Basic and applied research on yeasts, including yeast-like organisms
*Fungal Biology*	Elsevier	Hybrid	Fungal biology, including oomycetes and slime moulds
*Fungal Biology and Biotechnology*	BioMed Central	Open access	Fungal bio(techno)logy, including basic and applied research
*Fungal Ecology*	Elsevier	Hybrid	Fungal ecology, including population dynamics and role in the ecosystem
*Fungal Genetics and Biology*	Elsevier	Hybrid	Fungal biology, including molecular biology/genetics
*Journal of Fungi*	MDPI	Open access	Medical mycology, fungal pathogens
*Medical Mycology*	Oxford University Press	Hybrid	Medical mycology, fungal pathogens
*Mycologia*	Taylor and Francis	Open access	Fungal biology, including systematics, ecology, biodiversity and phylogenetic relationships
*Mycosphere*	Guizhou Academy of Agricultural Sciences	Open access	Fungal biology, including lichens
*Mycoscience*	Springer	Hybrid	Fungal biology, including systematics, ecology, biodiversity and phylogenetic relationships
*Applied Microbiology and Biotechnology*	Springer	Hybrid	Biotechnology, including biotech-relevant enzymes
*Biotechnology and Bioengineering*	Wiley	Hybrid	Biotechnology (broad definition)
*Biotechnology for Biofuels*	BioMed Central	Open access	Biotechnology, production of biofuels and other bioproducts
*Biotechnology and Bioprocess Engineering*	Springer	Hybrid	Biotechnology, bioengineering (broad definition)
*Biotechnology Letters*	Springer	Hybrid	Biotechnology, with focus on reactions and biocatalysis
*Journal of Biotechnology*	Elsevier	Hybrid	Biotechnology, including bioprocess engineering
*Metabolic Cell Factories*	BioMed Central	Open access	Biotechnology, with focus on metabolic engineering of microorganisms
*Molecular Biotechnology*	Springer	Hybrid	Biotechnology, with focus on molecular methods
*Nature Biotechnology*	Springer	Hybrid	Biotechnology (broad definition)

Crucially, *Fungal Biology and Biotechnology* embraced gold open access from its start (i.e. the articles are free of subscription charges for readers and university libraries, whereas to cover the costs an article-processing charge, APC, is taken over by the authors or their funders/host institutions). Following the success of this publishing model, pushed forward by non-profit pioneers such as the Public Library of Science [9] and its endorsement by funding agencies and learned societies (see e.g. the European Commission’s Horizon2020 goal on open access [10], the Berlin Declaration [11] or the San Francisco Declaration on Research Assessment [12]), coupled with the increasing criticism of the classic closed-access model dominated by few big players [13, 14], most journals offer an “open-access option.” This hybrid model (Table 1), with journals selling subscriptions while collecting APCs from authors choosing the open-access option for their article, is colloquially referred as “double-dipping” and overall not serving the cause of the open-access movement. Embracing gold open access is not based on ideology or self-referential for a journal like *Fungal Biology and Biotechnology*: it has been repeatedly reported how open-access articles have more citations and impact than closed-access ones as assessed by article-related metrics [15].

Overall, open access publishing is inscribed in the broader movement of *open science*, which can be loosely defined as an effort to make scientific advances available and accessible to any interested person disregarding her/his academic status, institute affiliation, access to funding or resources, etc. This includes sharing of protocols, data, or software using devoted platforms or pre-prints (i.e. articles prior of peer-review) on specific repositories/archives like bioRxiv [16] or PeerJ Preprints [17]. This latter practice is gaining momentum in the life sciences [18] and is accepted by an increasing number of journals (including those by BioMed Central), which do not consider it as an exclusion criterion for manuscript submission. The grand aim of *open science*—which is in full agreement with our conviction as editors of *Fungal Biology and Biotechnology*—is to remove barriers preventing the advancing of scientific research and to counteract the current culture of hyper-competiveness in science in favour of a more collaborative system. The advancement of openness in science is increasingly discussed amongst scientists, academics, non-profit organizations, service providers, funders, and librarians, as for example during the 1st Open Science Fair held the past summer in Athens [19], the international Open Access Week (this year October 23rd–27th, 2017) [20] or the upcoming OpenCon 2017 (November 11th–13th, 2017 in Berlin) [21].

It is an exciting time for (fungal) science and science dissemination. While we see *Fungal Biology and Biotechnology* growing and maturing, we are excited to witness how the openness culture of this journal will serve the fungal community.
